# Room-Temperature Performance of Poly(Ethylene Ether Carbonate)-Based Solid Polymer Electrolytes for All-Solid-State Lithium Batteries

**DOI:** 10.1038/s41598-017-17697-0

**Published:** 2017-12-13

**Authors:** Yun-Chae Jung, Myung-Soo Park, Duck-Hyun Kim, Makoto Ue, Ali Eftekhari, Dong-Won Kim

**Affiliations:** 1 Hanyang University, Department of Chemical Engineering, Seoul, 04763 Republic of Korea; 2 Battery R&D center, Samsung SDI, Gyeonggi-do, 16678 Republic of Korea; 3 The Engineering Research Institute, Ulster University, Newtownabbey, BT37 OQB United Kingdom; 4 School of Chemistry and Chemical Engineering, Queen’s University Belfast, Stranmillis Road, Belfastt, BT9 5AG United Kingdom

## Abstract

Amorphous poly(ethylene ether carbonate) (PEEC), which is a copolymer of ethylene oxide and ethylene carbonate, was synthesized by ring-opening polymerization of ethylene carbonate. This route overcame the common issue of low conductivity of poly(ethylene oxide)(PEO)-based solid polymer electrolytes at low temperatures, and thus the solid polymer electrolyte could be successfully employed at the room temperature. Introducing the ethylene carbonate units into PEEC improved the ionic conductivity, electrochemical stability and lithium transference number compared with PEO. A cross-linked solid polymer electrolyte was synthesized by photo cross-linking reaction using PEEC and tetraethyleneglycol diacrylate as a cross-linking agent, in the form of a flexible thin film. The solid-state Li/LiNi_0.6_Co_0.2_Mn_0.2_O_2_ cell assembled with solid polymer electrolyte based on cross-linked PEEC delivered a high initial discharge capacity of 141.4 mAh g^−1^ and exhibited good capacity retention at room temperature. These results demonstrate the feasibility of using this solid polymer electrolyte in all-solid-state lithium batteries that can operate at ambient temperatures.

## Introduction

Lithium-ion batteries (LIBs) have rapidly become the dominant power sources for portable electronic devices because of their long cycle life and high energy density, and their applications have expanded to electric vehicles (EVs) and energy storage systems (ESSs)^1–7^. However, safety issues still prevent the full utilization of such batteries due to the fact that LIBs use flammable and volatile liquid electrolytes. Thus, enhancing the safety of LIBs has become a significant concern, especially in large-capacity applications such as EVs and ESSs. For this reason, all-solid-state lithium batteries with solid electrolytes have been developed in attempts to improve the safety of conventional LIBs that employ liquid electrolytes^8–17^. Among the various types of solid electrolytes, solid polymer electrolytes present a variety of potential advantages, including their non-flammability, absence of solvent leakage, chemical stability, good interfacial contact with electrodes, low cost, easy processing, good film formability and flexibility in the shape of the battery design^15–18^. Since poly(ethylene oxide) (PEO)-based solid polymer electrolytes were first reported in 1973, they have been intensively investigated for battery applications^18–23^. However, their low ionic conductivities at ambient temperatures preclude their practical applications for use in lithium batteries at room temperature. Additionally, PEO has a relatively low dielectric constant, indicating that it is unable to fully dissociate lithium salts, resulting in the formation of un-dissociated lithium salt and ion agglomerations in solid polymer electrolytes^24–26^. Moreover, their poor mechanical properties resulting from their melting transition at high temperatures may cause short circuits between two electrodes if unexpected heat is generated^27^. As an alternative to PEO-based solid polymer electrolytes, polycarbonate-based solid polymer electrolytes have been studied due to their amorphous nature and the high dielectric constant of carbonates for effectively dissolving lithium salts^28–34^. Sun *et al*. reported poly(trimethyl carbonate)-based polymer electrolytes. However, their ionic conductivities were lower than 10^−8^ S cm^−1^ at room temperature, thus, cells assembled with this polymer electrolyte could only be operated at a very low current rate (1/55 C)^28,29^. Tominaga’s group reported solid polymer electrolytes based on commercially available poly(ethylene carbonate) (PEC), which showed high ionic conductivity and favorable lithium transference number at room temperature^30–33^. However, they could not be applied to rechargeable lithium batteries without a supporting membrane due to their poor dimensional stability^32^. Organic-inorganic hybrid solid electrolytes based on poly(ethylene oxide-co-ethylene carbonate) and octa-aminopropyl polyhedral oligomeric silsesuioxane were prepared and applied to solid-state lithium batteries^34^. However, the solid-state lithium batteries assembled with V_2_O_5_ cathode material could only be operated at high temperatures (~60 °C).

In this study, we synthesized poly(ethylene ether carbonate) (PEEC) via ring-opening polymerization of ethylene carbonate. This material showed an amorphous structure with a low glass transition temperature. Solid polymer electrolytes were then prepared with PEEC and lithium salt by a solution casting method, and their electrochemical properties were investigated. In order to improve the mechanical strength of the polymer electrolyte, a three-dimensional cross-linked polymer electrolyte was synthesized by photo cross-linking reaction using tetraethyleneglycol diacrylate (TEGDA) as a cross-linking agent. The cross-linked solid polymer was applied to the all-solid-state lithium cells composed of a lithium anode and a layered LiNi_0.6_Co_0.2_Mn_0.2_O_2_ cathode, and their electrochemical performance was evaluated at ambient temperatures.

## Results and Discussion

**Figure 1:**
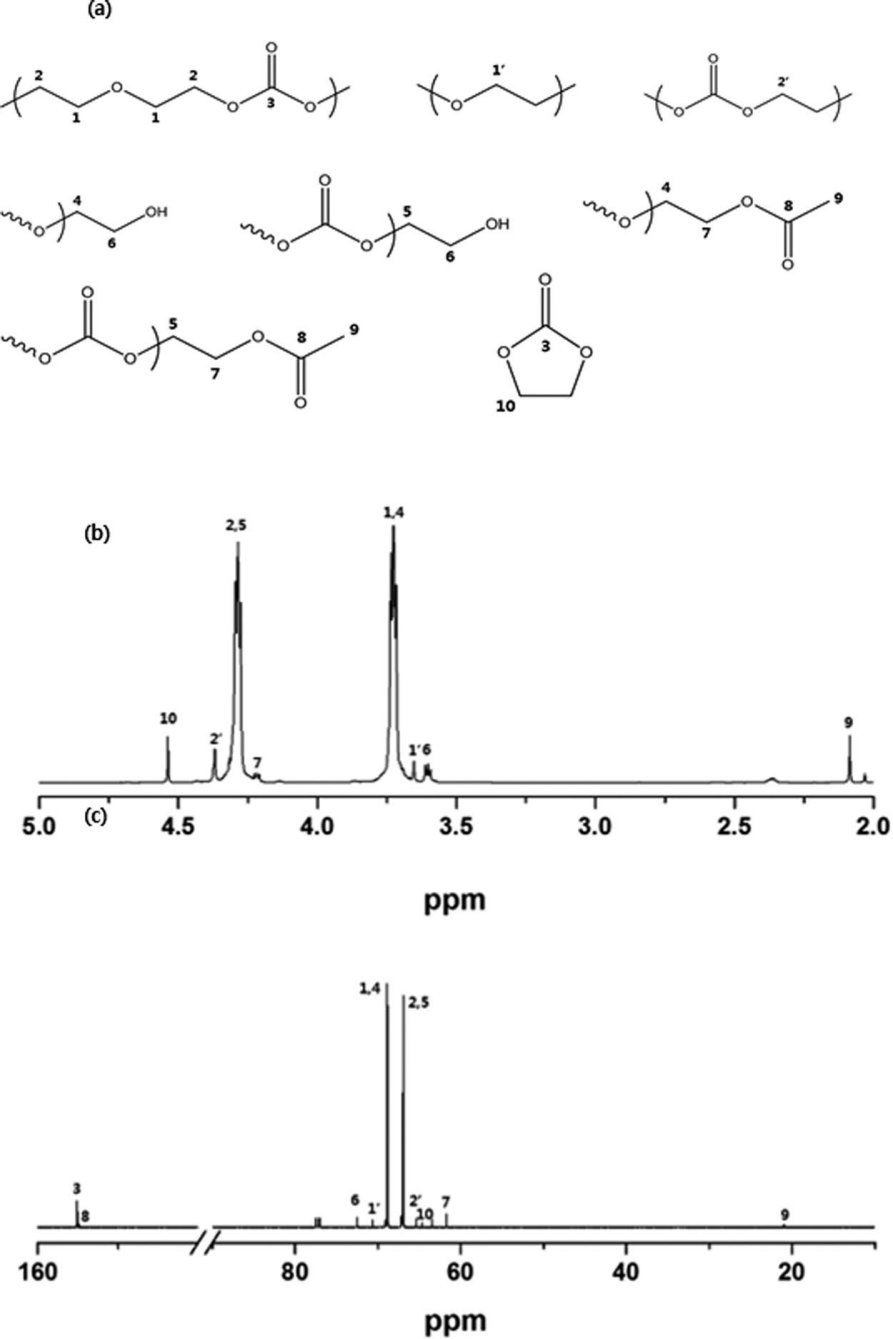
(**a**) Chemical moieties in PEEC. (**b**) ^1^H and (**c**) ^13^C NMR spectra of PEEC in CDCl_3_ at 25 °C.

**Figure 2:**
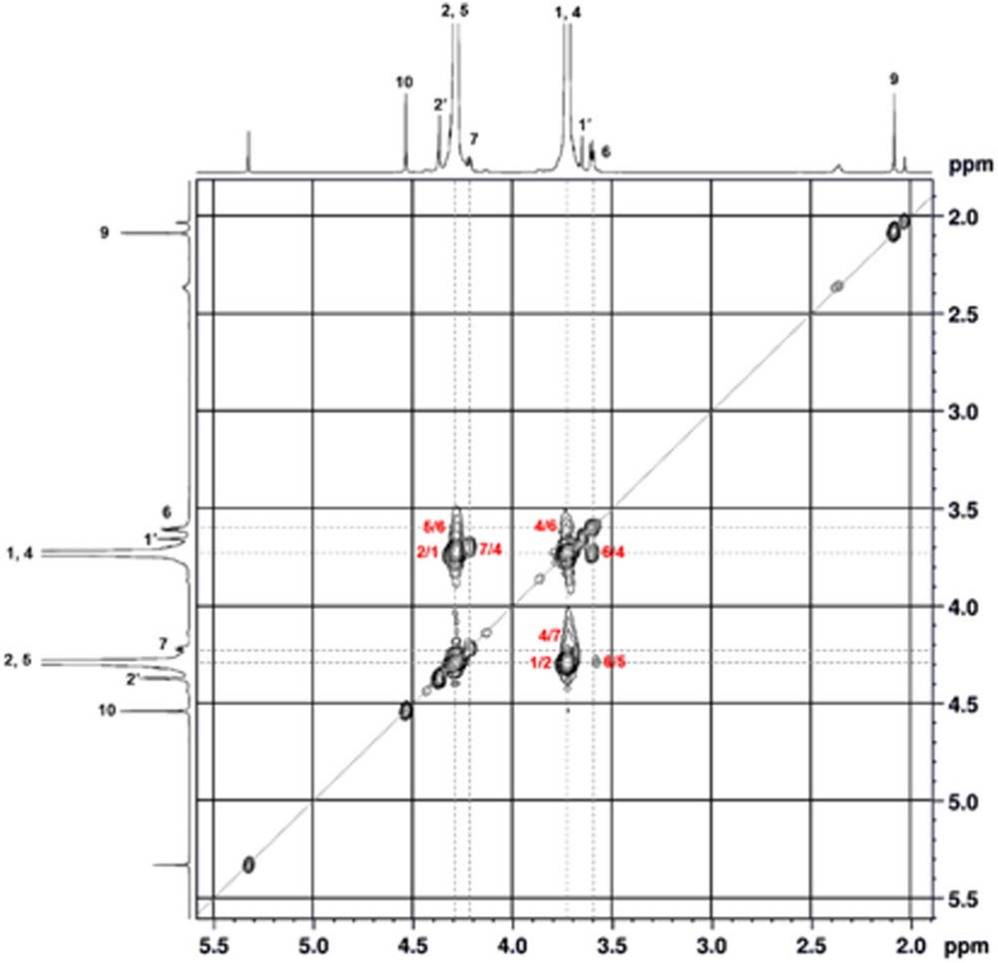
Correlation spectroscopy (COSY) spectrum of poly(ethylene ether carbonate).

The chemical structure of PEEC was characterized by analyzing its ^1^H and ^13^C NMR spectra, and the peak assignments were performed using two-dimensional (2D) NMR spectroscopy. Figure 1 shows the ^1^H and ^13^C NMR spectra of PEEC, with the peak assignments. In the ^1^H NMR spectrum of PEEC, the main peaks at 4.29 and 3.73 ppm were be assigned to the protons adjacent to the carbonate unit and the ether oxygen, respectively^34^. This confirms the presence of both ethylene carbonate and ethylene oxide units in the synthesized polymer. The molar ratio between the ethylene carbonate and ethylene oxide units in PEEC could be calculated from the integration ratio of the corresponding proton peaks (ethylene carbonate: 1,4 and ethylene oxide: 2,5) in Fig. 1b. As a result, the molar ratio of the ethylene carbonate and ethylene oxide unit was 49.5:50.5. In the ^13^C NMR spectrum of Fig. 1c, the two strong carbon peaks observed at 68.0 and 67.1 ppm could be assigned to the carbon adjacent to the ether oxygen and the carbonate group, respectively. Correlation spectroscopy (COSY) and heteronuclear single-quantum correlation spectroscopy (HSQC) were used to identify the detailed structure of PEEC. The resulting spectra are shown in Figs 2 and S1, respectively. In the COSY spectrum of PEEC (Fig. 2), the cross peaks of 2/1, 5/6, 7/4 and 4/6 are clearly shown. From the cross peak of 2/1 that has the strongest intensity, it is confirmed that ethylene oxide and ethylene carbonate share a vicinal coupling. Molar composition and average molecular weights of PEECs obtained at different reaction conditions are summarized in Table 1. Based on the data in this table, the PEECs obtained at different reaction conditions showed an almost same molar ratio between the ethylene oxide and ethylene carbonate. GPC results show that the average molecular weight of PEEC decreases as the amount of catalyst increases and the reaction time decreases.

**Table 1:** Synthesis condition and physical characteristics of poly(ethylene ether carbonate)s.

Sample	Reactant composition [catalyst:monomer] (molar ratio)	Reaction time (h)	Polymer composition [EO:EC](mol %)	M_n_	M_w_	PDI
PEEC1	1:100	24	49.5:50.5	3141	4179	1.33
PEEC2	1:100	48	48.3:51.7	3368	4695	1.39
PEEC3	1:100	72	48.5:51.5	3948	6060	1.53
PEEC4	2:100	24	49.8:50.2	3069	4122	1.34
PEEC5	2:100	48	49.9:50.1	3267	4358	1.33
PEEC6	2:100	72	49.9:50.1	3343	4537	1.36

**Figure 3:**
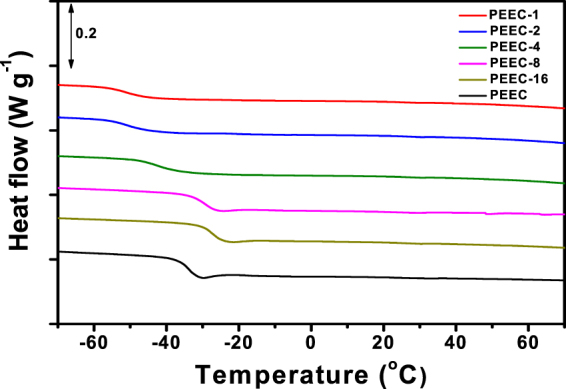
DSC thermograms of PEEC and PEEC-based polymer electrolytes with different salt concentrations.

DSC analysis was performed to investigate the thermal behavior of PEEC and PEEC-based polymer electrolytes, and the results are shown in Fig. 3. The salt concentration in the polymer electrolyte is expressed as a molar ratio of ([EO]+[EC])/[LiTFSI], as given in Table 2. In the DSC thermogram of PEEC without lithium salt, no melting transition peak was observed, indicating that PEEC is an amorphous polymer. The glass transition temperature (T_g_) of PEEC was measured to be −34 °C, which means that the synthesized PEEC is a flexible rubbery polymer with high segmental motion at ambient temperatures. It can be confirmed that the T_g_ value of PEEC lies between those of PEO (−64 °C) and PEC (9 °C). By adding lithium bis(trifluoromethane) sulfonylimide (LiTFSI) salt into PEEC at a molar ratio ([EO]+[EC])/[LiTFSI] of 16, the T_g_ value slightly increased. This result is due to the physical cross-linking effect, which results from a strong interaction between ether oxygen atoms and lithium ions^35^. However, the T_g_ values of PEEC-based polymer electrolytes decreased when the concentration of lithium salt was further increased (from PEEC-16 to PEEC-1). This behavior can be explained by the increased conformational mobility of the polymer backbone that occurs with increasing salt concentrations, as has been previously reported^30^. Moreover, the plasticizing of host polymer with an addition of TFSI anion is also attributed to the decrease in T_g_ values of polymer electrolytes. From thermogravimetric analysis (TGA) (Fig. S2), it is confirmed that the PEEC-based polymer electrolyte is thermally stable up to about 180 °C.

**Table 2:** Composition of PEEC and PEO-based solid polymer electrolytes.

Polymer electrolyte	PEEC or PEO (wt.%)	TEGDA (wt.%)	LiTFSI (wt.%)	([EO]+[EC])/ [Li^+^]
PEEC-1	19		81	1/1
PEEC-2	32		68	2/1
PEEC-4	48		52	4/1
PEEC-8	65		35	8/1
PEEC-16	79		21	16/1
XPEEC-1	17	10	73	1/1
PEO-16	71		29	16/1

**Figure 4:**
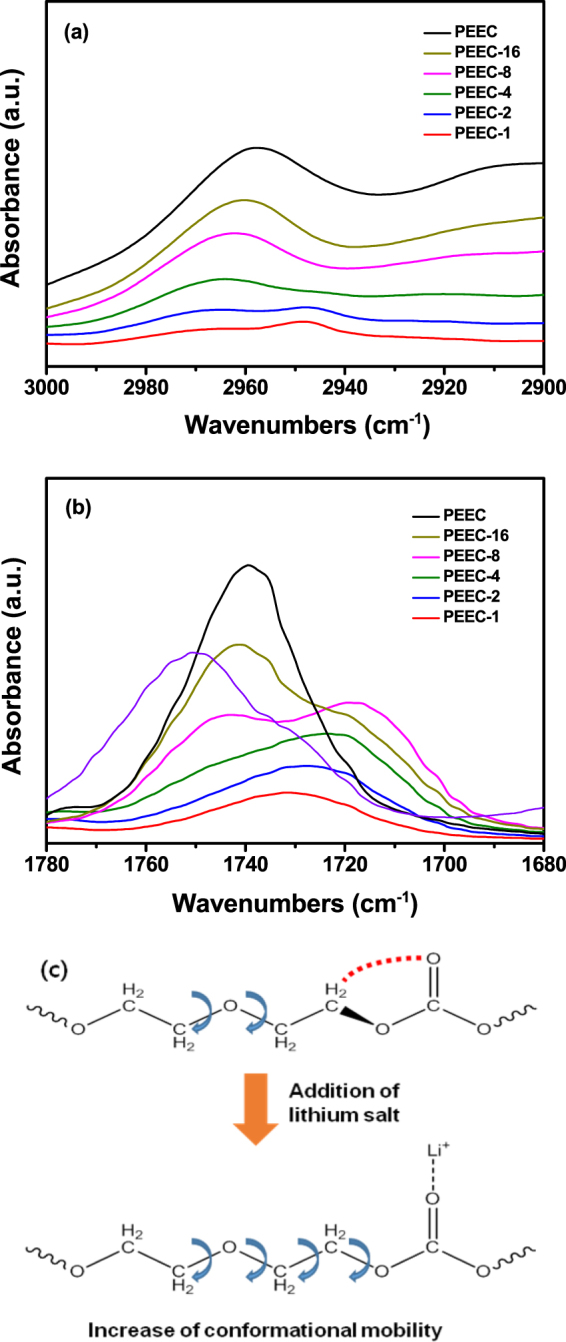
FT-IR spectra of PEEC and PEEC-based polymer electrolytes in the range of (**a**) 2900–3000 cm^−1^ and (**b**) 1680–1780 cm^−1^. (**c**) Schematic showing the increased conformational mobility of PEEC upon the addition of lithium salt.

To investigate the chain conformation of the PEEC containing lithium salt in the polymer electrolyte, FT-IR analysis was performed. The band observed at 2960 cm^−1^ for neat PEEC in Fig. 4a can be assigned to the CH_2_ stretching vibration of the gauche conformation of the C-C bond on the PEEC main chain. As the salt concentration increases, a new band appears at 2950 cm^−1^, which corresponds to the CH_2_ stretching vibration of normal alkanes. The band at 1740 cm^−1^ of neat PEEC in Fig. 4b can be identified as the stretching vibration of the carbonyl group on the PEEC main chain. The interacting band appears below 1720 cm^−1^ when the salt concentration is increased. These results indicate that the local rotational motion of the PEEC chain is enhanced by the addition of lithium salt, as schematically illustrated in Fig. 4c. Therefore, the T_g_ values of PEEC-based polymer electrolytes decreased with increasing salt concentrations, and the dissociated ions could migrate faster as a result of the improved segmental motion at high salt concentrations. Enhanced segmental motion with increasing salt concentrations in the poly(ethylene carbonate)-based polymer electrolytes has also been reported by Tominaga’s group^30–33,36^. An increase in the intensity of band at 1720 cm^−1^ with increasing salt concentration suggests that PEEC can dissolve a large amount of lithium salt.

**Figure 5:**
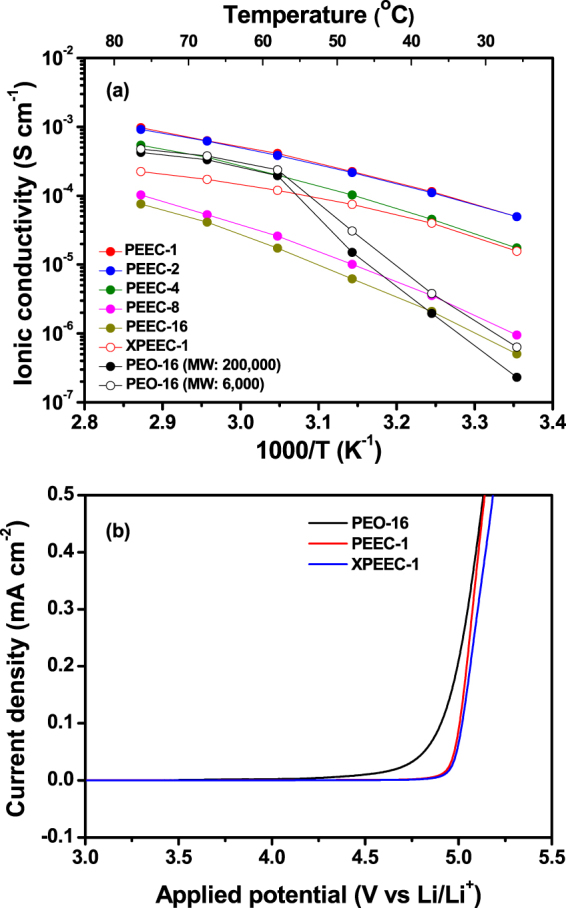
(**a**) Temperature dependence of ionic conductivities of various solid polymer electrolytes with different compositions, and (**b**) linear sweep voltammograms of PEO and PEEC-based solid polymer electrolytes at 55 °C (scan rate: 1 mV s^−1^).

The ionic conductivity of the polymer electrolyte was determined by ac impedance measurements using the cells with blocking electrodes. Figure 5a shows the temperature dependence of the ionic conductivities for the PEEC-based polymer electrolytes with different salt concentrations. The ionic conductivities of the PEO-based polymer electrolytes (MW of PEO: 6,000 and 200,000) are also shown for comparison. As expected, the PEO-based solid polymer electrolytes exhibited low ionic conductivities at ambient temperatures. Note that use of PEO with low molecular weight (MW: 6,000) slightly increases the ionic conductivity due to higher ionic mobility by the enhanced segmental motion. However, it was difficult to prepare a free-standing film when using low molecular weight PEO. Thus, the PEO-based polymer electrolyte prepared with only high molecular weight PEO was considered as a control sample for further studies. Below melting transition temperature of PEO, the activation energies for ionic conduction in the PEO-based polymer electrolytes are relatively high due to its high degree of crystallinity. On the other hand, the PEEC-based polymer electrolytes showed higher ionic conductivities than the PEO-based polymer electrolytes at room temperature, and the ionic conductivity continuously increased with increasing salt concentrations, as depicted in Fig. S3. The higher ionic conductivities of the PEEC-based polymer electrolytes at ambient temperatures are believed to be due to the amorphous character of PEEC, as mentioned earlier in the DSC results, this is the case because high ionic conductivity is necessarily associated with the amorphous phase of the polymer^37^. Also, the more favorable dissociation of lithium salt in PEEC can be attributed to the higher ionic conductivity, which is due to the fact that the ethylene carbonate has a higher dielectric constant for dissolving salt than the ethylene oxide moiety^25,26^. As discussed in the DSC and FT-IR results, the addition of lithium salt into PEEC improves the segmental motion of polymer chain and decreases the glass transition temperature of the polymer electrolytes (Fig. S3). Thus, the increase in ionic conductivity with increasing salt concentration can be ascribed to both the increase in ionic mobility and the number of charge carriers. It was found that the temperature dependence of the ionic conductivities for the PEEC-based polymer electrolyte exhibited Vogel-Tamman-Fulcher (VTF) behavior throughout the temperature range investigated in this study, as has been reported in other amorphous polymer electrolytes^37,38^. This result suggests that the ionic conduction in PEEC mainly depends on the segmental motion of the polymer chain. Although the PEEC-based polymer electrolyte with high salt concentration (PEEC-1) exhibited high ionic conductivity at room temperature, it was difficult to handle due to its poor mechanical stability, thus, it could not be directly applied to the solid-state lithium cell. In order to improve the dimensional stability of the polymer electrolyte, a three-dimensional cross-linked polymer electrolyte was synthesized via photo cross-linking reaction using PEEC-1 and TEGDA as a cross-linking agent. The resulting solid polymer electrolyte was in the form of a freestanding flexible and rubbery thin film, as depicted in Fig. S4. The thickness of the cross-linked solid polymer electrolyte film ranged from 60 to 100 μm. As shown in Fig. 5a, the cross-linking of PEEC caused a slight decrease in the ionic conductivity compared to that of the non-cross-linked polymer electrolyte (PEEC-1). The ionic conductivity of the cross-linked polymer electrolyte (XPEEC-1) is 1.6 × 10^−5^ S cm^−1^ at room temperature, which is still two orders of magnitude higher than that of the PEO-based polymer electrolytes. The electrochemical stability of various polymer electrolytes was evaluated by linear sweep voltammetric (LSV) measurements at 55 °C, and the resulting LSV curves are shown in Fig. 5b. It should be noted that LSV measurements were performed at 55 °C, because the ionic conductivity of PEO-based polymer electrolyte (PEO-16) was too low to measure oxidative current at ambient temperature. As shown in the figure, the oxidative current started to increase around 4.5 V vs. Li/Li^+^ in the PEO-based polymer electrolyte, which can be attributed to the oxidative decomposition of PEO. In contrast, the PEEC-based polymer electrolyte (PEEC-1) exhibited electrochemical stability above 4.9 V, indicating that introducing the carbonate units into the polymer backbone improved the oxidative stability of the polymer electrolyte. Furthermore, this electrochemical stability is better than other solid polymer electrolytes such as poly(vinyl carbonate)^39^. The cross-linking of the PEEC-based polymer electrolyte by TEGDA hardly affected the oxidative stability of the cross-linked polymer electrolyte (XPEEC-1). Based on these results, the PEEC-based cross-linked polymer electrolyte exhibits higher ionic conductivity and electrochemical stability than the PEO-based solid polymer electrolyte, which makes it suitable for application in a solid-state Li/LiNi_0.6_Co_0.2_Mn_0.2_O_2_ cell.

**Figure 6:**
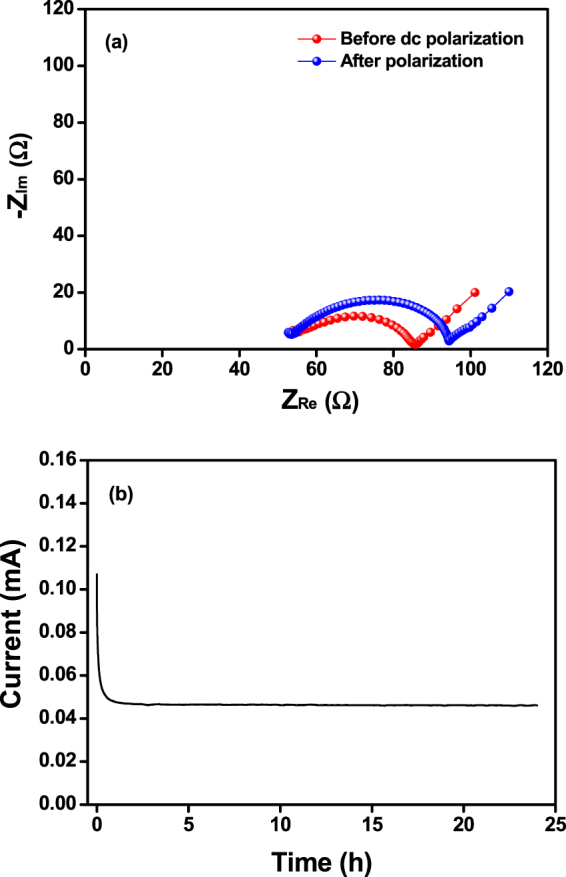
(**a**) AC impedance spectra of a Li/XPEEC-1/Li cell at 55 °C before and after dc polarization. (**b**) Variation of the current as a function of time during dc polarization in a Li/XPEEC-1/Li cell at 55 °C with an applied potential difference of 20 mV.

The lithium transference number (t^+^) in the PEEC-based cross-linked polymer electrolyte (XPEEC-1) was measured by a combination of ac impedance and dc polarization measurements^40,41^. The ac impedance measurement of the Li/XPEEC-1/Li cell (Fig. 6a) was used to determine the initial interfacial resistance. A small dc potential was then applied to the cell, and the current was monitored as a function of time until a steady-state current was established, as depicted in Fig. 6b. The steady-state interfacial resistance of the cell was again determined via an ac impedance measurement, as shown in Fig. 6a. From the data in Fig. 6a and b, the lithium transference number in XPEEC-1 was calculated to be 0.40, indicating that the mobility of the Li^+^ ions is slightly lower than that of the anions. This is due to the fact that the Li^+^ ions are strongly coordinated by the polymer chains through ion-dipole interactions, while the anions are loosely associated with the polymer segments, allowing them to be displaced more readily under an electric field. It is noticeable that the value of t^+^ in XPEEC-1 was much higher than one (0.16) measured in the PEO-based polymer electrolyte. As Tominaga *et al*. previously reported, the migration of Li^+^ ions can be decoupled from the segmental dynamics in the ethylene carbonate unit, resulting in an increased lithium transference number^30^. These results suggest that the introduction of ethylene carbonate units into the polymer backbone can increase the lithium transference number in the polymer electrolyte.

**Figure 7:**
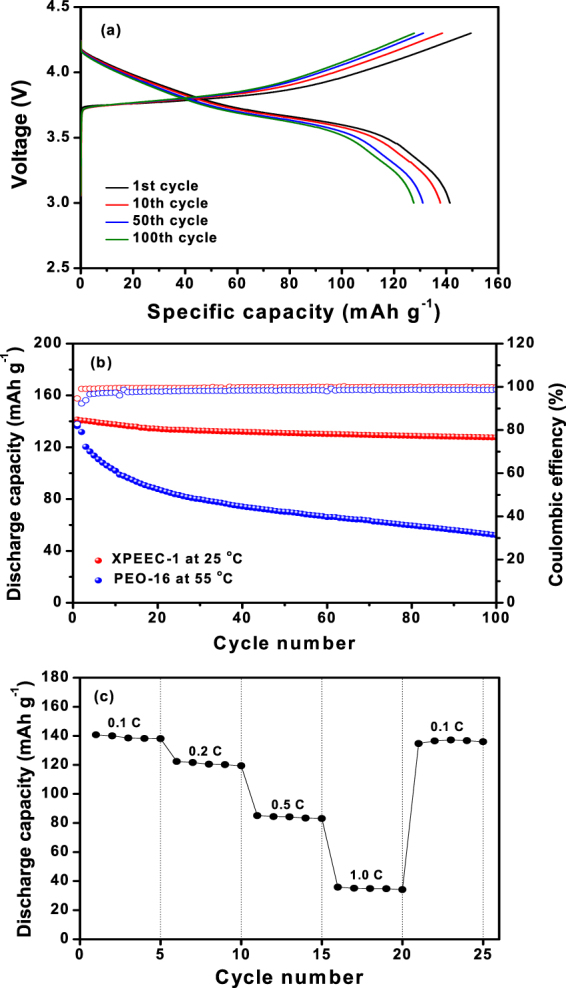
(**a**) Charge/discharge curves of the Li/XPEEC-1/LiNi_0.6_Mn_0.2_Co_0.2_O_2_ cell, (**b**) discharge capacities of Li/LiNi_0.6_Mn_0.2_Co_0.2_O_2_ cells assembled with XPEEC-1 and PEO-16 as a function of the cycle number (0.1 C, cut-off voltage: 3.0–4.3 V, 25 °C for XPEEC-1, and 55 °C for PEO-16), and (**c**) discharge capacities of Li/XPEEC-1/LiNi_0.6_Mn_0.2_Co_0.2_O_2_ cell as a function of C rate.

A solid-state Li/LiNi_0.6_Co_0.2_Mn_0.2_O_2_ cell was assembled with XPEEC-1, and its cycling performance was evaluated. Figure 7a shows the charge and discharge curves of the Li/LiNi_0.6_Co_0.2_Mn_0.2_O_2_ cell assembled with cross-linked PEEC electrolyte at 25 °C. The cell delivered an initial discharge capacity of 141.4 mAh g^−1^, based on the LiNi_0.6_Co_0.2_Mn_0.2_O_2_ material in the positive electrode. The coulombic efficiency was initially 94.8%, which steadily increased with cycling to reach a constant value of over 99.5% throughout cycling after the initial few cycles. Figure 7b shows the discharge capacities and coulombic efficiencies of the cells assembled with the XPEEC-1 (at 25 °C) and PEO-based electrolytes (at 55 °C), respectively. It should be noted that the cell with PEO-based polymer electrolyte could not operate at room temperature due to the high resistance of the polymer electrolyte. The discharge capacity of the cell with XPEEC-1 decreased from 141.4 mAh g^−1^ to 127.6 mAh g^−1^ at the 100th cycle, which corresponds to 90.2% of the initial discharge capacity. In contrast, the discharge capacity of the cell assembled with the PEO-based electrolyte decreased from an initial discharge capacity of 136.2 mAh g^−1^ to 52.2 mAh g^−1^ at the 100th cycle, corresponding to 38.3% of the initial value. It is noticeable that the cell assembled with XPEEC-1 electrolyte exhibited higher discharge capacity and coulombic efficiency than the cell with the PEO-based electrolyte throughout cycling. The enhanced cycling performance of the cell with the cross-linked PEEC electrolyte is caused by the higher lithium transference number and better electrochemical stability. The high t^+^ value in the polymer electrolyte results in high lithium ion conductivity and less polarization of the cell potential^41^. With respect to the cycling stability, a layer-structured cathode material, such as LiNi_0.6_Co_0.2_Mn_0.2_O_2_, can easily oxidize the oxyethylene group in the PEO-based polymer electrolyte. Accordingly, the cell with the PEO-based polymer electrolyte exhibited a large capacity decline and low coulombic efficiency, which was caused by the oxidative decomposition of PEO at high voltage during repeated charge-discharge cycles^42^. In the cell assembled with XPEEC-1, the carbonate unit in the PEEC backbone and high salt concentration can improve the oxidative stability of the solid polymer electrolyte. Super-concentrated electrolytes with enhanced electrochemical stability have also been reported by some research groups^43,44^. The highly-adhesive properties of the PEEC-based polymer electrolyte also allowed it to maintain good interfacial contact with the electrodes during the charge and discharge processes. These results suggest that using the cross-linked PEEC-based solid electrolyte enables all-solid-state lithium cells to operate at ambient temperatures. Figure 7c shows the discharge capacities of the Li/LiNi_0.6_Co_0.2_Mn_0.2_O_2_ cell assembled with XPEEC-1, during experiments in which the C rate was increased from 0.1 to 1.0 C every five cycles. The discharge capacities gradually decreased as the C rate was increased, thereby demonstrating polarization. Thus, both ionic conductivity of polymer electrolyte and lithium ion diffusivity in the positive electrode should be further improved to obtain good rate capability. More systematic studies related to the optimization of the solid polymer electrolyte and the proper design of electrodes suitable for PEEC-based solid polymer electrolytes are currently in progress in attempts to improve the cycling performance of all-solid-state lithium batteries.

## Conclusion

Here we reported a novel solid polymer electrolytes based on PEEC as an alternative to the common PEO-based solid electrolytes for all-solid-state lithium battery. Owing to the amorphous nature of the polymer matrix, the Li^+^ ions have a higher degree of mobility as can be judged from high ionic conductivity and transference number. As a result, the solid electrolyte can successfully perform at the room temperature, which is a major issue in the battery application of solid polymer electrolytes. The presence of ethylene carbonate units within the polymer backbone facilitates the transport of Li^+^ ions and widens the stable potential window. The solid polymer electrolyte was successfully used in the fabrication of a cell made of Li anode and LiNi_0.6_Co_0.2_Mn_0.2_O_2_ cathode, which exhibited good cycling performance at room temperature. All-solid-state Li/LiNi_0.6_Co_0.2_Mn_0.2_O_2_ cell delivered a high initial discharge capacity of 141.4 mAhg^−1^ and exhibited good capacity retention with high coulombic efficiency at 25 °C, thereby demonstrating room temperature operation of this solid-state lithium cell using a solid polymer electrolyte.

## Methods

### Materials

Ethylene carbonate (EC, battery grade, PANAX ETEC Co. Ltd.), lithium bis(trifluoromethane) sulfonylimide (LiTFSI, battery grade, PANAX ETEC Co. Ltd.), triethylene glycol diacrylate (TEGDA, Sigma-Aldrich), 2-hydroxy-2-methylpropiophenone (HMPP, TCI), tetrahydrofuran (THF, HPLC-grade, Sigma-Aldrich) and N-methyl-2-pyrrolidone (NMP, Sigma-Aldrich) were used as received. Poly(ethylene oxide) (PEO, M_w_ = 6,000 and 200,000) was purchased from Sigma-Aldrich and used after vacuum drying at room temperature for 3 days. Dibutyltin diacetate (DBTDA, TCI) was dried under vacuum at 80 °C for 24 h before use. Acetonitrile (anhydrous, Alfa Aesar) and methanol were used as received.

### Synthesis of poly(ethylene ether carbonate) (PEEC)

PEEC was synthesized via ring-opening polymerization of ethylene carbonate using DBTDA as a catalyst, as shown in Fig. S5. Polymerization was performed under an argon flow at 165 °C. A 250-ml three-neck flask charged with 50.0 g (0.567 mol) of EC and DBTDA was placed in an oil bath. After polymerization, the polymer was purified by filtration though a glass frit funnel to remove insoluble catalyst residues, and the filtrate was dissolved in chloroform. This was followed by precipitation in an excess of methanol. The methanol layer was decanted, and the oily residue was rinsed several times with methanol. The polymer dissolved in dichloromethane was eluted though a silica gel column. After solvent evaporation, the transparent and yellowish PEEC could be obtained as a final polymer product.

### Preparation of PEEC-based polymer electrolytes

Appropriate amounts of PEEC and LiTFSI were dissolved together in an anhydrous acetonitrile solvent. The salt concentration in the polymer electrolytes was expressed as a molar ratio of ([EO]+[EC])/[LiTFSI], as given in Table 2. The solution was stirred well and cast on a Teflon plate using a doctor blade. The solvent was then allowed to slowly evaporate at room temperature. The resulting film was further dried in a vacuum oven at 60 °C for at least 24 h. In order to prepare the cross-linked polymer electrolyte, 10 wt.% of TEGDA and a catalytic amount of HMPP (0.2 wt.% of TEGDA) were added into the above solution, these served as a cross-linking agent and photo-initiator, respectively. The cast mixture on a Teflon plate was exposed to UV light (254 nm) for 10 min in order to induce the photo cross-linking reaction. The resulting cross-linked polymer electrolyte was dried under vacuum at 80 °C for 24 h. All of the preparation procedures were performed in a glove box filled with argon gas.

### Characterizations

^1^H and ^13^C NMR spectra were recorded in CDCl_3_ with a tetramethylsilane (TMS) reference using an Avance-500 MHz NMR spectrometer (Bruker) at room temperature. The attenuated total reflection Fourier transform infrared (ATR-FTIR) spectra of PEEC and PEEC-based polymer electrolytes were obtained on a Nicolet iS50 Fourier transform infrared spectrometer (Thermo Scientific) in the wavenumber range of 400 to 4000 cm^−1^. The average molecular weights and polydispersity indices (PDIs) of PEEC polymers were measured by gel permeation chromatography (GPC, Waters 1515) equipped with three columns in series (i.e., Styragel® HR 1 THF, Styragel® HR 4E THF and Styragel® HR 5E THF). The system with a refractive index (RI) detector was calibrated using polystyrene standards. HPLC-grade THF was used as an eluent. Differential scanning calorimetry (DSC) measurements were carried out to examine the thermal transition behavior of the PEEC polymer and PEEC-based polymer electrolytes using a TA instrument (SDT Q600/DSC Q20) at a heating rate of 5 °C min^−1^ in the temperature range from −80 to 80 °C under a dry nitrogen atmosphere. TGA was performed using a TGA analyzer (SDT Q600, TA Instrument) in the temperature range from 30 to 500 °C at a heating rate of 10 °C min^−1^.

### Electrode preparation and cell assembly

**Figure 8:**
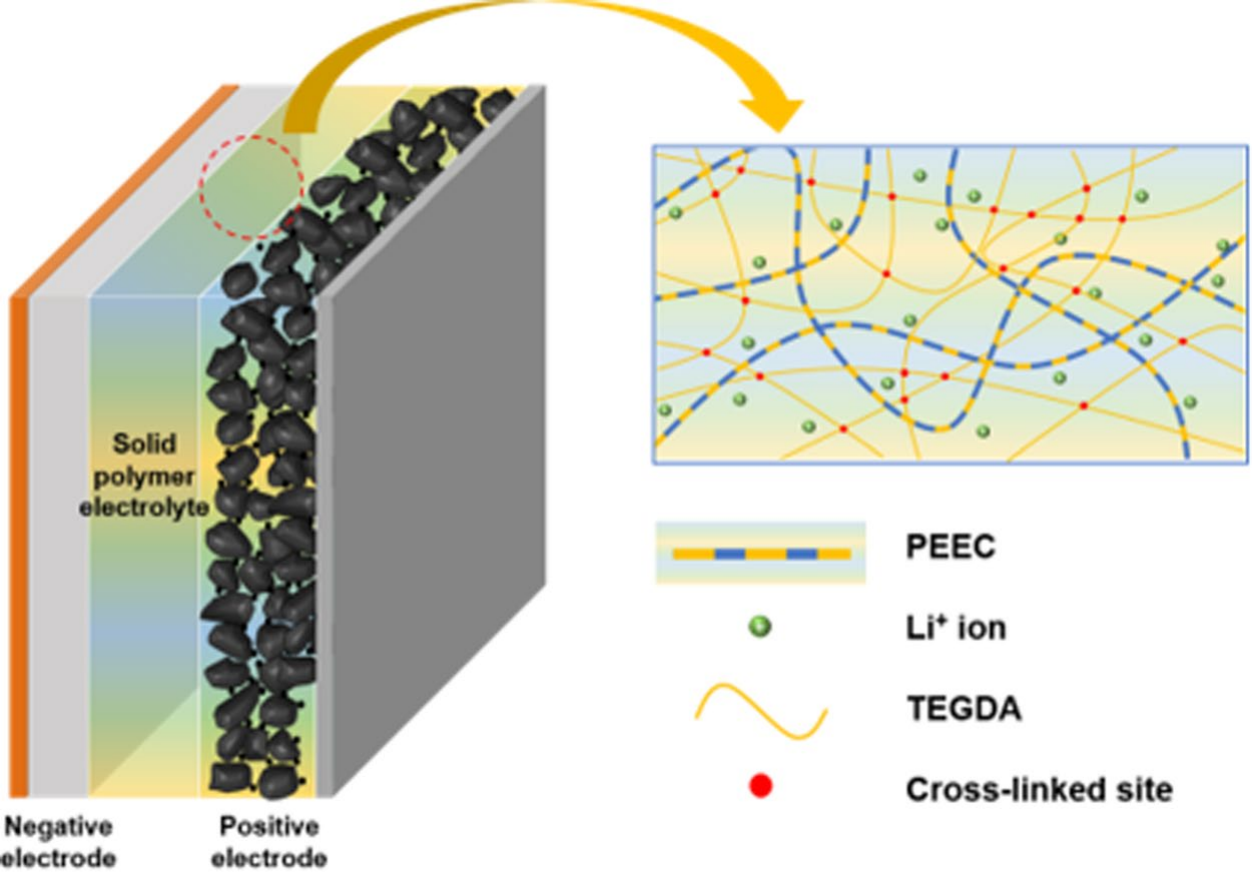
Schematic presentation of all-solid-state Li/LiNi_0.6_Co_0.2_Mn_0.2_O_2_ cell assembled with cross-linked PEEC-based solid polymer electrolyte.

The composite positive electrode was prepared by coating an NMP-based slurry containing LiNi_0.6_Co_0.2_Mn_0.2_O_2_, PEEC, LiTFSI, poly(vinylidene fluoride) (PVdF) and Super P carbon (70: 1.86: 8.14: 5: 15 by weight) onto Al foil. PEEC was used as a Li^+^ ion conductor as well as a binder in the composite positive electrode. The electrode was dried under vacuum for 12 h at 110 °C and then roll pressed to enhance particulate contact and adhesion to the current collector. The active mass loading in the positive electrode was about 4.2 mg cm^−2^. The negative lithium electrode consisted of a 200-μm-thick lithium foil (Honjo Metal Co., Ltd.) that was pressed onto a copper current collector. A solid-state Li/LiNi_0.6_Co_0.2_Mn_0.2_O_2_ cell was a ssembled by sandwiching the solid polymer electrolyte (XPEEC-1 or PEO-16) between the negative lithium electrode and the positive LiNi_0.6_Co_0.2_Mn_0.2_O_2_ electrode, as schematically presented in Fig. 8. After cell assembly, the cells were kept at 55 °C for 24 h in order to promote interfacial contact between the solid polymer electrolyte and the positive LiNi_0.6_Co_0.2_Mn_0.2_O_2_ electrode. All of the cells were assembled in a glove box filled with argon gas.

### Electrochemical measurements

For ionic conductivity measurements, solid polymer electrolytes were sandwiched between two disk-like stainless steel electrodes. AC impedance measurements were carried out using a Zahner Electrik IM6 impedance analyzer over the frequency range from 10 Hz to 100 kHz with an amplitude of 10 mV at different temperatures. Each sample was allowed to equilibrate for 1 h at the required temperature before measurement. Linear sweep voltammetry (LSV) experiments were performed to investigate the electrochemical stability of the polymer electrolytes on a platinum working electrode, with counter and reference electrodes of lithium metal, at a scanning rate of 1.0 m V s^−1^ and 55 °C. The lithium ion transference number (t^+^) in the solid polymer electrolytes was measured in the Li/solid polymer electrolyte/Li cell by using a combination of ac impedance and dc polarization measurements at 55 °C^40,41^. For interfacial resistance measurements, solid polymer electrolyte was sandwiched between two lithium electrodes and sealed in coin cells. AC impedance measurements were performed in the frequency range from 100 mHz to 100 kHz at 55 °C. Charge and discharge cycling tests of the solid-state Li/LiNi_0.6_Co_0.2_Mn_0.2_O_2_ cells were conducted at a constant current rate of 0.1 C in the voltage range of 3.0 to 4.3 V using battery test equipment (WBCS 3000, Wonatech) at 25 °C.

## Electronic supplementary material

Supplementary Information
