# Supplementary material for: Room-Temperature Performance of Poly(Ethylene Ether Carbonate)-Based Solid Polymer Electrolytes for All-Solid-State Lithium Batteries
Source: Sci Rep. 2017 Dec 13;7:17482. doi: 10.1038/s41598-017-17697-0 (PMC5727542; doi:10.1038/s41598-017-17697-0)
Supplement: Supplementary file 1 — Supplementary Information [file 41598_2017_17697_MOESM1_ESM.pdf]

Supplementary Information

Room-Temperature Performance of Poly(Ethylene Ether Carbonate)-Based Solid Polymer Electrolytes for All-Solid-State Lithium Batteries

Yun-Chae Jung[1], Myung-Soo Park[1], Duck-Hyun Kim[2], Makoto Ue[2], Ali Eftekhari[3,4] & Dong-Won Kim[1,*]

[1]Department of Chemical Engineering, Hanyang University, Seoul 04763, Republic of Korea

[2]Battery R&D Center, Samsung SDI, Gyeonggi-do 16678, Republic of Korea

[3]The Engineering Research Institute, Ulster University, Newtownabbey BT37 OQB, United Kingdom

[4]School of Chemistry and Chemical Engineering, Queen's University Belfast, Stranmillis Road, Belfast BT9 5AG, United Kingdom

*dongwonkim@hanyang.ac.kr

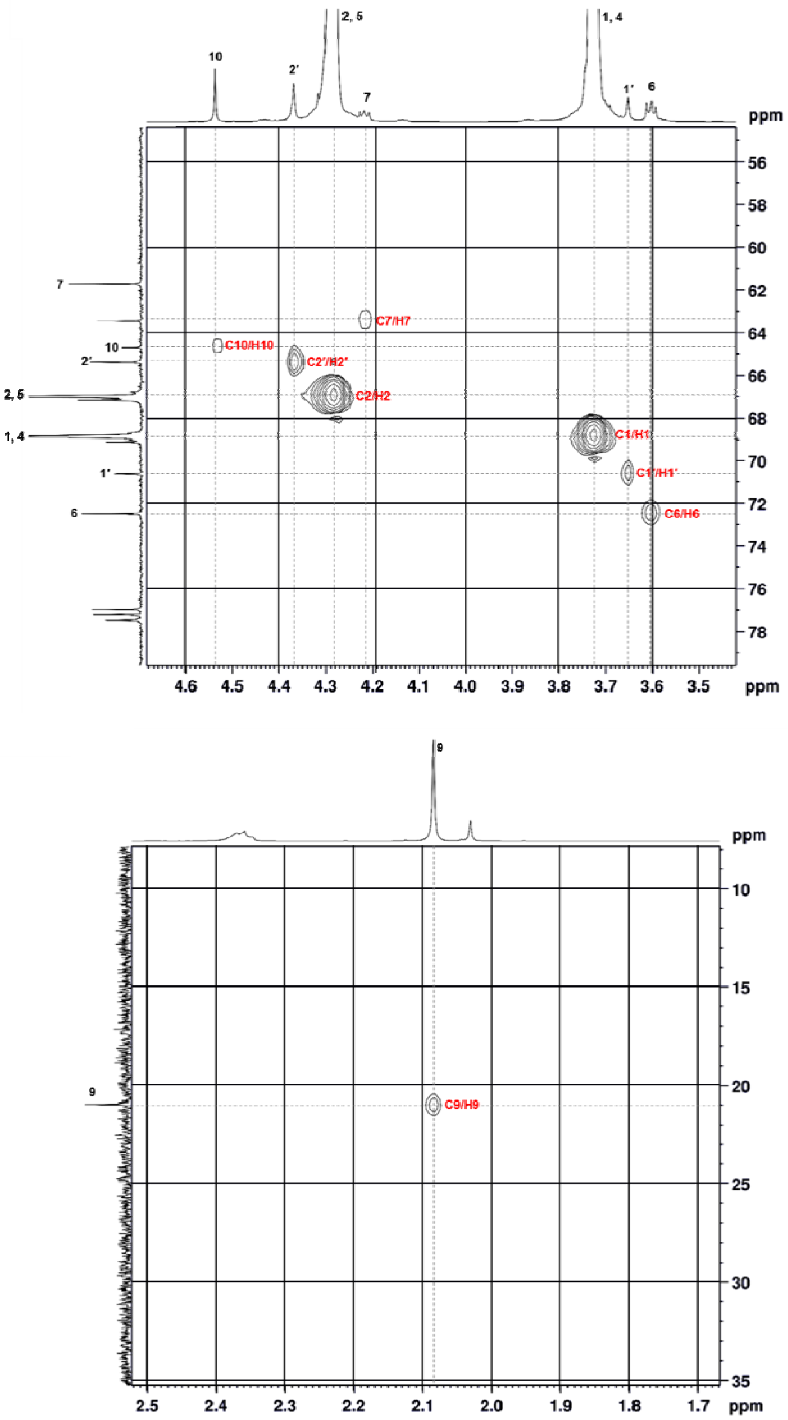

Figure S1. Heteronuclear single-quantum correlation spectroscopy spectra of PEEC.

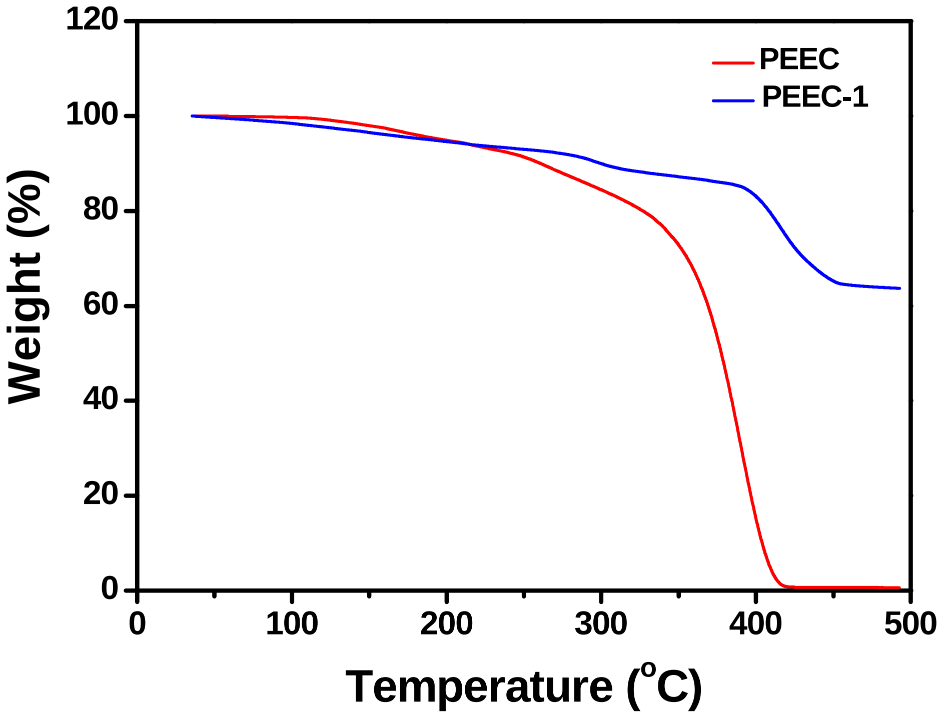

Figure S2. TGA curves of PEEC and PEEC-based polymer electrolyte (PEEC-1).

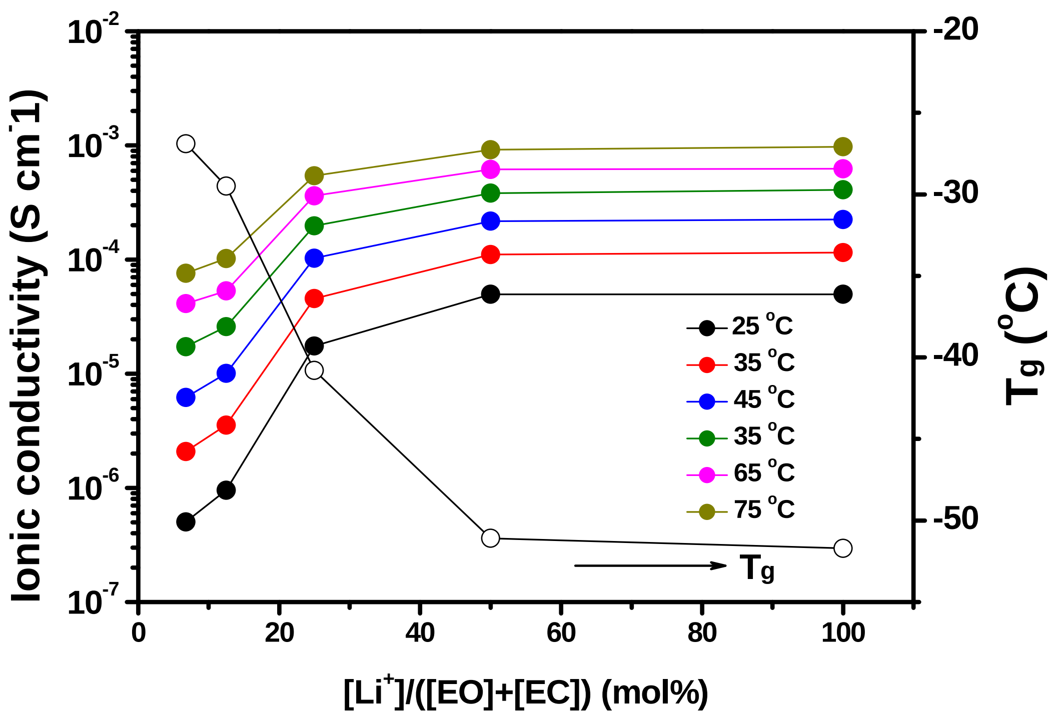

Figure S3. Ionic conductivities and glass transition temperatures of PEEC-based polymer electrolytes as a function of salt concentration.

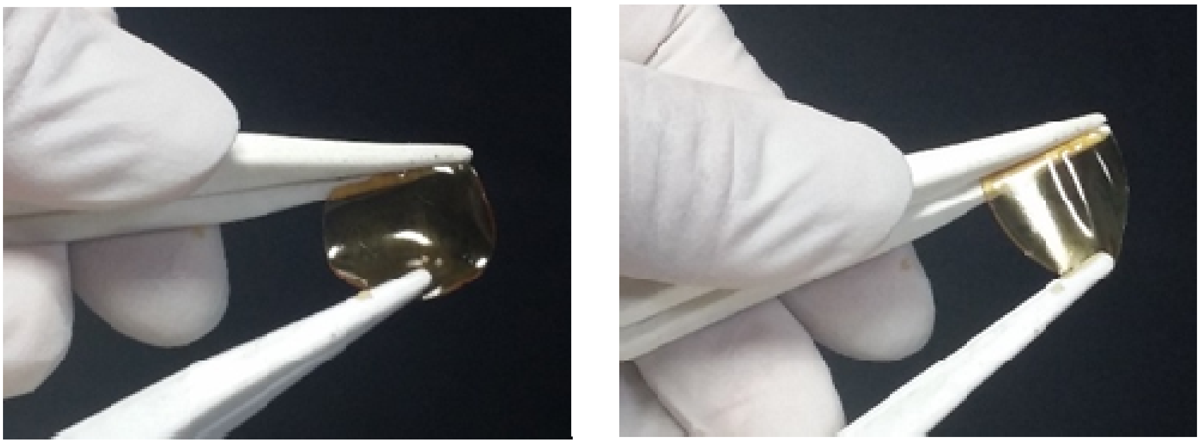

Figure S4. Photographs of cross-linked PEEC-based solid polymer electrolyte film.

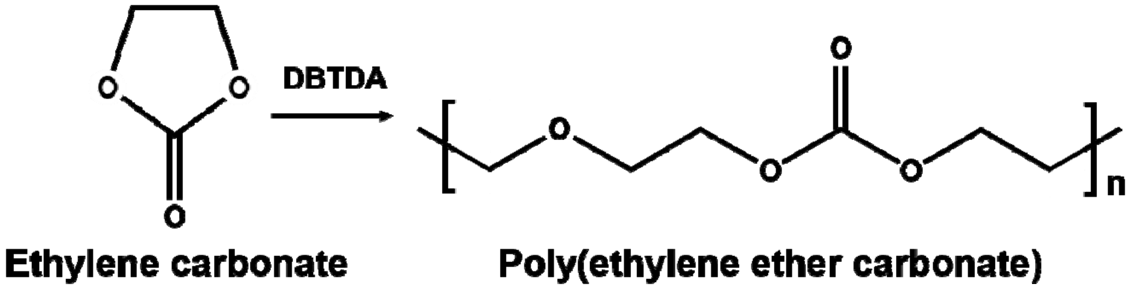

Figure S5. Synthesis of poly(ethylene ether carbonate) from ethylene carbonate via ring-opening polymerization.
